# A Transesophageal Echocardiogram Finding: From Infection to Malignancy

**DOI:** 10.7759/cureus.6886

**Published:** 2020-02-05

**Authors:** Omar Kousa, Janani Baskaran, Aiza Ahmad, Dana H Awad, Ahmed Aboeata

**Affiliations:** 1 Internal Medicine, CHI Creighton University Medical Center, Omaha, USA; 2 Internal Medicine, Creighton University, Omaha, USA; 3 Cardiology, Creighton University, Omaha, USA

**Keywords:** libman-sacks endocarditis, malignancy, infection

## Abstract

Nonbacterial thrombotic endocarditis (NBTE) is illustrated by thrombi deposition on normal heart valves without the presence of bacteremia. It typically occurs in the setting of chronic debilitating diseases such as cancer or autoimmune disease. The pathogenesis involves an endothelial injury in the presence of a hypercoagulable state secondary to the effects of circulatory cytokines, which triggers platelet deposition. It usually forms on the upstream atrial surface of the mitral and tricuspid valves and the ventricular surface of the pulmonic and aortic valves and occurs most commonly in the fourth to eighth decades of life with no specific gender predisposition. These vegetations have a distinct morphology that varies from infective endocarditis (IE). Cerebrovascular lesions due to NBTE have a distinctive pattern of multiple, widely distributed small and large strokes on brain magnetic resonance imaging (MRI). We present a case of a 78-year-old man who was initially diagnosed as pneumonia and IE; he underwent a trans-esophageal echocardiogram (TEE), which revealed Libman-Sacks findings that have changed his diagnosis to lung cancer. We aim to highlight the characteristic TEE findings of NBTE to help clinicians search for underlying etiologies, including malignancies if NBTE is suspected.

## Introduction

Libman-Sacks valvular lesions are sterile, fibro-fibrinous vegetations that mostly occur on the left-sided heart valves and usually form on the ventricular surface of the aortic and pulmonic and the atrial surface of the mitral and tricuspid valves [1-2]. The pathogenesis is thought to involve the formation of fibrin-platelet thrombi, which organizes and leads to fibrosis and scarring with subsequent valve dysfunction. These vegetations have a distinct morphology that varies significantly from infective endocarditis. The overall clinical picture of the patient’s symptoms, cultures, and imaging are crucial to reach the right diagnosis and determine the subsequent management. We present a case of advanced malignancy diagnosed based on a transesophageal echocardiogram (TEE) showing vegetations suspicious for nonbacterial thrombotic endocarditis (NBTE).

## Case presentation

**Figure 1:**
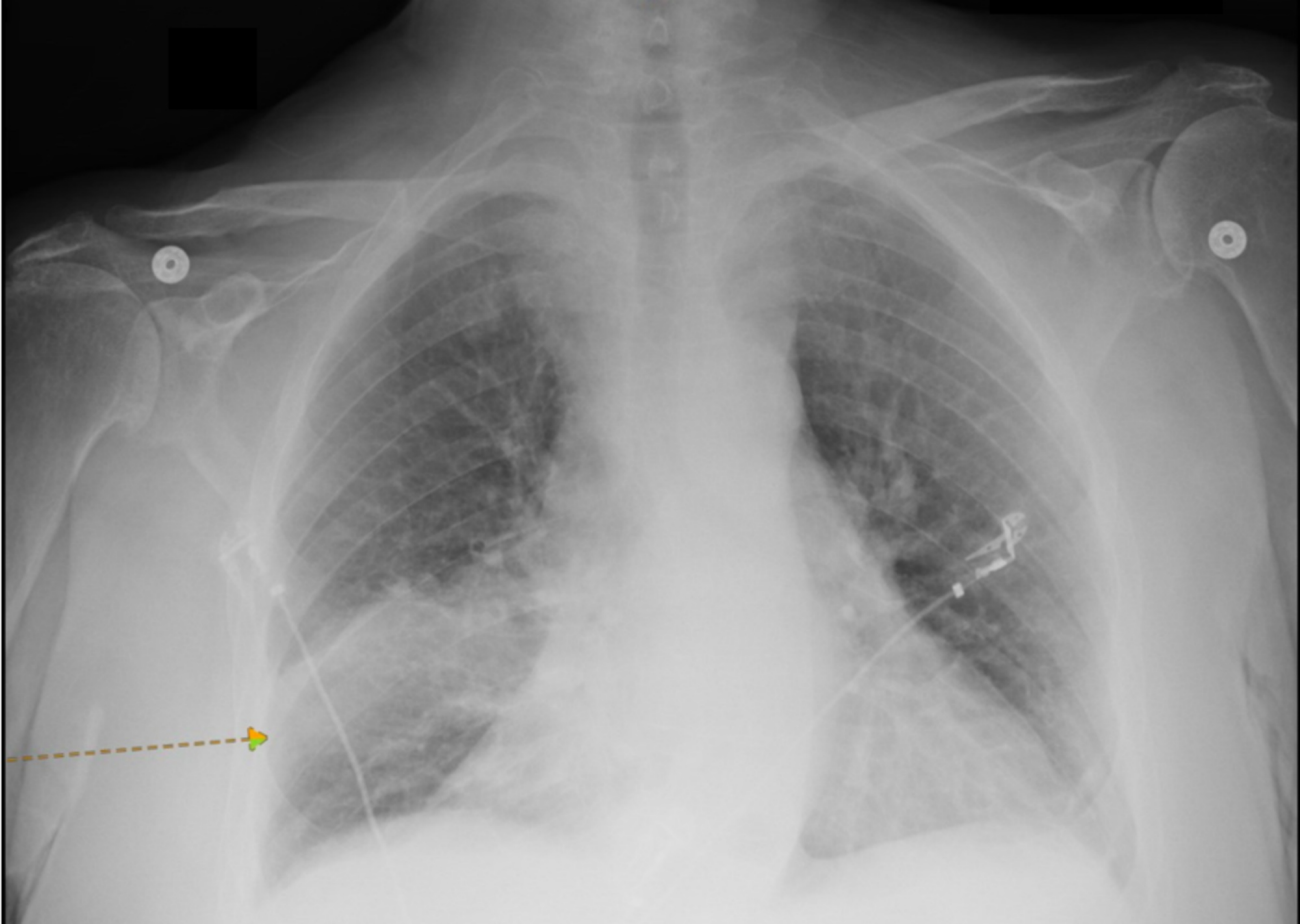
Chest X-ray There is opacity in the right lower lung (arrow).

A 78-year-old Caucasian man with a history of permanent left side muscle weakness due to a previous poliovirus infection was brought to the emergency department from a nursing home because of hypoxia. He had shortness of breath and productive cough for one week. His oxygen saturation on room air was found to be at 84% at the nursing facility. He was recently diagnosed with deep vein thrombosis and was started on apixaban. He had a history of hypertension, for which he was on losartan and metoprolol, hyperlipidemia on atorvastatin, and diabetes mellitus on glimepiride. On physical exam, he was afebrile, respiratory rate of 16 breath per minute, heart rate of 90 beats per minute, oxygen saturation 96% on room air, and blood pressure of 132/75 mmHg. Chest examination revealed bilateral end-expiratory wheezes, and the neurological examination showed no facial asymmetry, gaze, aphasia, or sensory loss. However, left upper and lower limb power was 3/5 as compared to 5/5 on the right side with no change from his baseline. Laboratory tests showed an elevated white blood cell count of 21.4 thousand cells per microliter with absolute neutrophils (83%). Electrocardiogram and arterial blood gas were within normal. The diagnosis of pneumonia was made based on a chest radiograph showing right basilar opacity (Figure 1), and he was started on broad-spectrum antibiotics. A transthoracic echocardiogram was done the following day, showing an average left ventricle ejection fraction of 60% to 65% with grade 1 diastolic dysfunction and left ventricular hypertrophy with no significant valvular pathology. The patient’s blood culture was positive for coagulase-negative staphylococci in one out of the two bottles, and he underwent a TEE, which showed poorly defined multiple subtle echo densities on the atrial side of the posterior leaflet of the mitral valve and on the ventricular side of the aortic valve mostly at the coaptation edges with no significant valvular damage suggestive of sterile vegetations. (Videos 1-2).

**Video 1:** Multiple subtle echo densities on the atrial side of the posterior leaflet of the mitral valve

**Video 2:** Multiple subtle echo densities on the ventricular side of the aortic valve mostly at the coaptation edges

**Figure 2:**
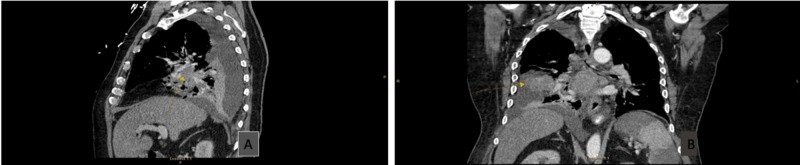
CT chest A: sagittal view; B: coronal view; Both show mass-like consolidation in the posterior right middle lobe with abnormal enhancement, suspicious for neoplasm (arrows)

**Figure 3:**
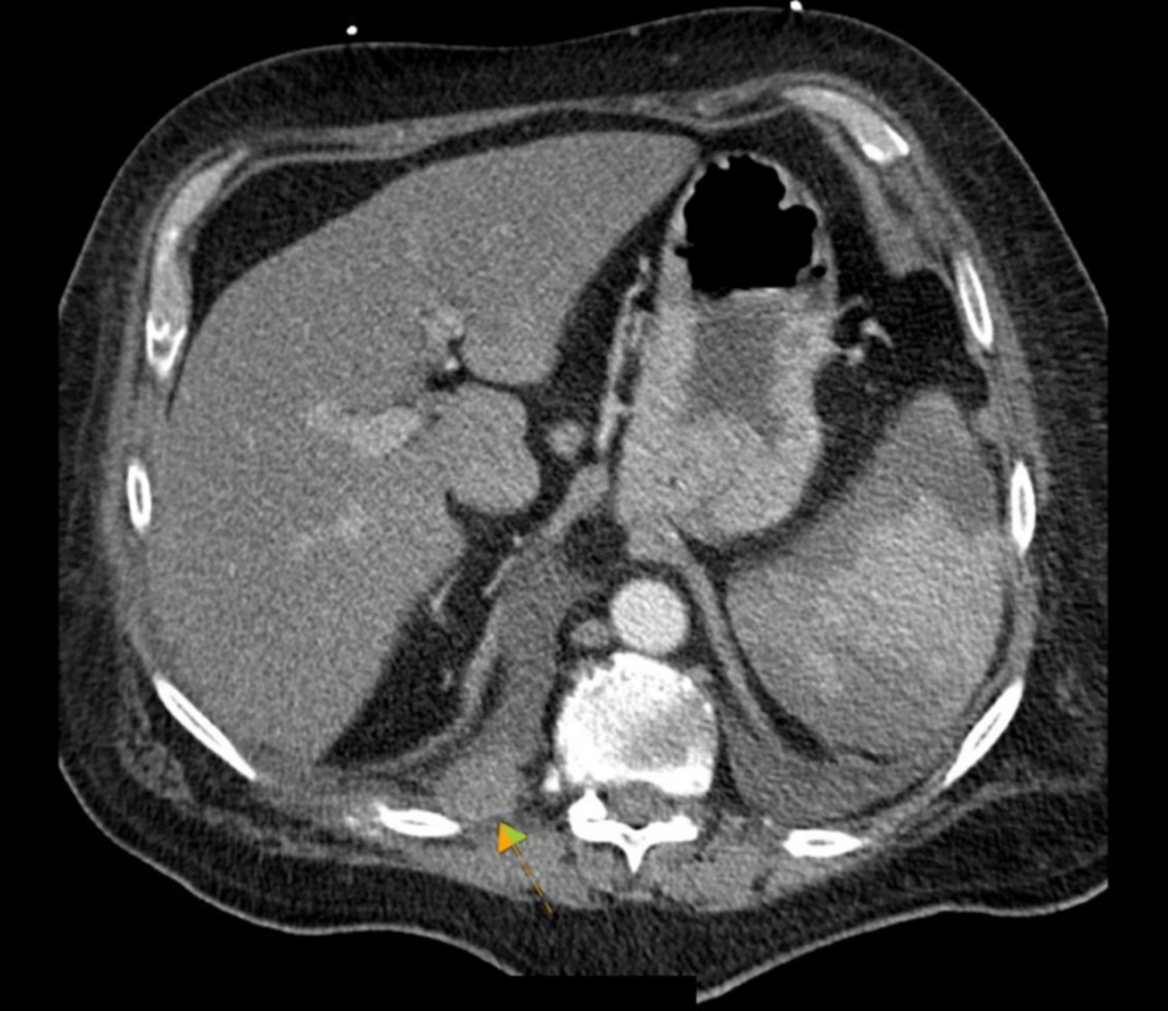
CT chest axial view Right pleural soft tissue nodules, compatible with pleural metastasis (arrow)

Given his pneumonia and bacteremia, infective endocarditis was initially considered, and a plan for a prolonged course of antibiotics was elected. However, the morphological appearance of the valve lesions on the TEE sparked concern for NBTE. Given his recent deep venous thrombosis and lung consolidation on chest radiograph, the cardiologist advised checking for underlying malignancy. Computed tomography of the chest showed a mass-like consolidation in the posterior right lobe with evidence of metastasis with multiple bony lytic lesions and pleural metastasis (Figures 2-3). Blood cultures were subsequently determined to be contamination. The patient and his family decided not to proceed with any further investigations and opted for comfort care measures only.

## Discussion

Endocarditis is an inflammation of the heart valve and endocardium, which is characterized by the formation of vegetation, and it can be bacterial or NBTE. Pre-existing valve damage was the most common predisposing factor, as the most common valvular lesions found in males were cardiac valvular dystrophic sclerocalcifications, whereas post-rheumatic valve lesions were found in females [3]. The incidence and prevalence of NBTE are unclear since the diagnosis of NBTE is most commonly made at autopsy. One study reported a 19% prevalence of NBTE in patients with malignancy, diagnosed by echocardiography - 10 times more than the control group (without overt heart disease) [4]. NBTE occurs most commonly in the fourth to eight decades of life, with no specific gender predisposition [5-7]. Whereas another study found 405 cases out of a total of 10,874 autopsies, representing 3.7% of cases. Malignant neoplasms were found in 59%, and thrombosis was found in 38% of NBTE cases [3]. NBTE is characterized by thrombi deposition on normal heart valves without the presence of a bloodstream infection, and it typically occurs in the setting of chronic debilitating diseases such as cancer and autoimmune disorders. The aortic valve is most commonly affected, followed by the mitral valve [7-8]. The vegetations also have a unique location - the atrial surface of the mitral and tricuspid valves and the ventricular surface of the aortic and pulmonary valves - and are found on the coapting edges of the leaflets and do not alter or impede the function of the valve.

The pathogenesis involves an endothelial injury in the presence of a hypercoagulable state. The endothelial damage is thought to be caused by circulatory cytokines (tumor necrosis factor or Interleukin-1), which triggers platelet deposition. In particular, the presence of an underlying condition where the coagulation system is activated (malignancy, disseminated intravascular coagulation, or antiphospholipid antibody syndrome) results in the deposition of platelets and inflammatory molecules. The vegetation is called “white thrombi” since it consists of agglutinated blood and platelet thrombi immune complexes, mononuclear cells interwoven with fibrin strands, without signs of inflammation [9]. The size varies from microscopic to large vegetations, which is known as Verrucous or Libman Sacks endocarditis. The lack of an inflammatory reaction at the site of attachment makes these vegetations more prone to embolization than the vegetations in bacterial endocarditis. The incidence of embolization varies between 14% and 91%, with an average of 42% [5]. A six-year cross-sectional study amongst 76 systemic lupus erythematosus (SLE) patients showed that the presence of NBTE was an independent predictor for the development of neuropsychiatric SLE, with an odds ratio of 13.4, and patients with vegetations had three times more cerebral microembolic events per hour compared to those without vegetations [10].

Unlike bacterial endocarditis, with its many pathognomonic symptoms and signs, clinical manifestations of NBTE are rather rare. Clinical manifestations of NBTE result mostly from systemic emboli rather than valvular dysfunction. Murmurs are rarely heard and fevers are uncommon, much unlike Bacterial endocarditis in which about 50% of patients present with embolic phenomena [7,11]. Embolization to the central nervous system presents as stroke or delirium, embolization to the kidney presents as hematuria with or without flank pain, and embolization to skin presents as a rash. Pulmonary embolism is also common in NBTE although causation is difficult to establish since most of these patients have underlying malignancy or another hypercoagulable state [7]. NBTE should be suspected in any patient with multiple strokes, hypercoagulable state, and known or suspected malignancy.

Interestingly, the patterns of stroke vary from NBTE vs. infective endocarditis. NBTE has a uniform pattern of multiple, widely distributed small and large strokes while patients with IE have a variety of patterns on diffusion-weighted imaging magnetic resonance imaging. However, the size of the vegetations did not correlate with the pattern or number or size of strokes [12].

In this case, the patient was admitted because of pneumonia based on the radiological findings on his chest radiograph and the subsequent growth of his blood culture. His TEE showed valvular vegetations, which was initially correlated to his underlying bacteremia, and a prolonged course of antibiotic therapy was chosen. However, the distinct morphologic appearance of the vegetations and his recent diagnosis of deep venous thrombosis lead to further exploration of underlying malignancy, which changed the diagnosis, course of his hospitalization, and management options.

## Conclusions

Although most NBTE cases are found on autopsies, the recognition of distinctive TEE findings of NBTE can be the initial clue of an underlying malignancy. NBTE should be strongly suspected in any patient with a hypercoagulable state and known or suspected cancer. Further testing for underlying malignancy should be pursued even if blood cultures are positive when NBTE is suspected on echocardiography.
